# Integrated care systems in England: the significance of collaborative community assets in promoting and sustaining health and wellbeing

**DOI:** 10.3389/fsoc.2024.1355215

**Published:** 2024-08-06

**Authors:** Oonagh Corrigan, Scott Danielsen, Shannon Doherty, Pauline Lane

**Affiliations:** ^1^School of Allied Health and Social Care, Faculty of Health, Medicine, and Social Care, Anglia Ruskin University, Chelmsford, United Kingdom; ^2^Health, Partnerships and Wellbeing, Colchester City Council, Colchester, United Kingdom; ^3^THEME Institute, Colombo, Sri Lanka

**Keywords:** integrated care systems, community assets, complexity theory, health inequalities, community

## Abstract

Until recently the healthcare system in England was based on a commissioning/provider model. However, this has been replaced with an Integrated Care Systems (ICSs) approach, aimed at improving health and wellbeing and reducing inequalities through local collaborative partnerships with public sector organizations, community groups, social enterprise organizations and other local agencies. Part of this new approach is an emphasis on the role of community assets (i.e., local resources), that are considered integral to promoting positive health and wellbeing outcomes. This paper presents research from a series of three research studies on “community assets” conducted in the East of England within a newly established ICS. Based on analysis of qualitative data highlighting the lived experience of community asset members, this paper shows the positive wellbeing impact on vulnerable community members that assets provide. Further insight on the local impact and the collaborative nature of the research is provided suggesting that new asset-based approaches recognize the social determinants of health. This presents a shift away from positivistic linear approaches to population health and wellbeing to a new non-linear collaborative approach to addressing health inequalities and promoting wellbeing. The authors suggest that exploring this through a complexity theory lens could illuminate this further. Finally, the authors warn that while community assets have an important role to play in empowering citizens and providing much needed support to vulnerable and disadvantaged communities, they are not a substitute for functioning funded public sector services that are currently being undermined by ongoing local governments funding cuts. As such, while community assets can help ameliorate some of the negative effects people experience due to economic, structural and health disadvantages, only a more fair and more equal distribution of resources can address growing health inequalities.

## 1 Introduction

This paper presents findings from a series of three research studies conducted on “community assets” in the East of England within a newly established regional Integrated Care Systems (ICS). In accordance with the Health and Care Act (2022), ICSs were established across England between 2019 and 2023 as legal entities with statutory powers and responsibilities (Kings Fund, 2022). There are forty-two ICSs in England, and they are reputed to have brought about a fundamental shift in the way that health and care systems are organized and delivered, with an emphasis on collaboration, place-based partnership and bolstering local community assets (Goddard, 2023).

ICSs have been deploying new approaches to tackle growing regional inequalities by supporting community assets and ABCD (Asset Based Community Development). The NHS Ten Year Plan for England (NHS, 2019a) has placed an emphasis on preventing illness and tackling health inequalities. Part of that plan is to “do things differently” and it sets out an ambition to:

…*increase the focus on NHS organisations working with their local partners, as ‘Integrated Care Systems'*, [and] *plan and deliver services which meet the needs of their communities* (NHS, 2019b).

Community assets often involve a range of complex relationships, where different factors and people interact with each other in order to be responsive to peoples' needs in specific places (Thirsk and Clark, 2017). The general ABCD approach is seen as a shift away from approaches that previously focused on identifying prevailing social deficits to one where existing community assets are identified and supported; where communities are given a voice and enabled to grow and thrive. While recognizing that health inequalities arise because of structural inequalities in society (the conditions in which people are born, grow and live), in the present absence of a fairer economic redistribution of resources, community assets are seen as playing an important role in ameliorating some of the negative effects of growing inequalities (Marmot et al., 2013). Block (2018) and Russell (2020) have been highly influential protagonists in criticizing more traditional, top down, interventionist approaches to improving public health and argue that these are often ineffective and unsustainable. They contend that social change is not the result of individual behavioral change but emerges as a result of effective grassroot building at the community level and what is needed is a ground-up approach (Russell, 2020, p. 14). Block (2018) too is concerned with social transformation through fostering and building connected communities where citizens have a sense of belonging and social capital is promoted. These ideas have been informing local community government practices in the UK and elsewhere. For example, Nurture Development is a network providing ideas and practical support including training in promoting a shift to more place based, citizen centered approaches throughout the UK and beyond, influencing local government practices here and overseas (see Nurture Development, 2024). This shift represents a change in policy priorities away from a universal, non-context specific and traditional hierarchical expert-led approach, to one that highlights the importance of adopting place-based approaches and an increasing collaborative citizen/community centered approach to improve health and wellbeing outcomes (Charles et al., 2021).

Moreover, there is some evidence suggesting that community assets can have a positive health and wellbeing impact, helping to prevent some long-term conditions, and increase resilience and wellbeing in individuals and communities (Howarth et al., 2020). However, while they are often considered as an important means to tackle social deprivation and growing health inequalities, questions remain as to whether community assets can reduce health inequity in disadvantaged, marginalized or vulnerable communities (Cyril et al., 2015; Mughal et al., 2022).

In this paper we draw on the findings of three waves of research conducted between 2019 and 2022 in the East of England to reveal ways that community assets can and do improve wellbeing in vulnerable communities. Given that ICS and ABCD approaches involve more collaborative less linear hierarchical approaches to health and wellbeing, in this paper, we question whether “complexity theory” might further develop analysis of community assets as part of a wider system of collaborative integrated pluralistic approaches that gives appropriate weight to real-world case studies and embraces non-linear causality (Greenhalgh and Papoutsi, 2018).

### 1.1 Complexity theory

Complexity theory has been used to describe fluid, non-linear change within organizations and how they can adapt to their environments and manage conditions of uncertainty (Long et al., 2018). This theoretical perspective often emphasizes co-evolving, complex adaptive systems (Kauffman, 1993, 1995; Holland, 1995) and it has been influential in the field of organizational studies (Currie et al., 2012) and is being used to understand the contemporary healthcare landscape.

Conventional theories that seek to explain health and social care systems have traditionally been tethered to a quest for certainty, predictability, and linear causality. In contrast, complexity theory has started to be used to reveal the complexities of contemporary healthcare based on a recognition of the interrelated dynamics of the multiple components, systems and subsystems within wider socio-economic, and political spheres of healthcare (Apker, 2012; Greenhalgh and Papoutsi, 2018). As such, complexity theory may offer some useful insights into understanding the multiple dimensions of healthcare within the dynamics of ICSs. For example, Nason (2023) suggests that traditional knowledge about health has been held by professionals, and in the clinical setting, professional expertise is highly valued. However, knowledge about the determinants of health, and the context of health promotion and health equality, is held in many different domains, by professionals and ordinary citizens. By using the lens of complexity theory, we can see how community assets can be considered as part of the complex systems of healthcare involving communities in actively shaping their own development, through a process of co-production (Stutton, 2018).

When referring to health and or wellbeing we are using the terms broadly alluding to the relationship between the two where health relates to the physical and mental aspects broadly and where wellbeing suggests a positive state shaped by subjective feelings as well as social experiences (Daykin et al., 2021). The concept of wellbeing has multiple elements that transverse broad categories of emotion, behavior, cognition, and relationships. Adopting a complexity theory lens can help illuminate a well-used definition of wellbeing by exploring “how people feel and how they function both on a personal and social level, and how they evaluate their lives” (Michaelson et al., 2012, p. 6).

### 1.2 This research

The original research was commissioned by the North-East Essex Health and Wellbeing Alliance who are one of three place-based alliances within the Suffolk and North East Essex Integrated Care Partnership. The Alliance consists of a number of different organizations working in partnership to tackle specific community health issues, and to improve population health and wellbeing across the districts of Colchester and Tendring in North-East Essex (NEE-Alliance, 2020).

The commissioners were keen to understand how and to what extent community assets could help alleviate growing health inequalities and support those communities in socially deprived areas serving populations who are disadvantaged and/or vulnerable.

The region concerned can be characterized as complex, having a wide range of economic and social disparities, with some areas experiencing extreme social deprivation leading to increasing poor health and wellbeing outcomes (Essex County Council, 2019; Ministry of Housing, Communities and Local Government, 2019; Colchester Borough Council, 2020). Three waves of research were conducted between 2019 and 2022 (see Corrigan et al., 2020, 2021, 2023a,b). The first wave of research was conducted just prior to the first national COVID lockdown, the second wave immediately after lockdown restrictions were lifted and some community groups were resuming face-to-face activities, and the third when an emerging cost of living crisis was looming across the UK.

For the first wave of research the Alliance wished to improve their understanding of how local people's health and wellbeing was supported (or not), by “*community assets*”. Research findings were employed to inform their overall strategies to address inequalities and support ABCD. For subsequent waves, the Alliance wanted to know more about the resilience of the assets and the experiences of members during the and following the COVID pandemic, and in the context of an emerging cost of living crisis (Hill and Webber, 2022).

The research outlined in this paper can be considered as a new approach to community health and development insofar as the Alliance was seeking to work with local government public health officers, local charities and community groups to best understand how these assets work in practice. This was not a linear health intervention study but an example of co-production, as the researchers worked in collaboration with senior local government officers, commissioners, local community leaders, and members of the different community assets.

The aims of this research were 2-fold. (1) To provide a means for “vulnerable” citizens voices to be heard and to better understand people's “lived experiences” of those belonging to a community forum and engaging with a community asset. (2) To understand the impact of community assets and their effectiveness in supporting individuals' sense of wellbeing within the East of England.

## 2 Materials and methods

### 2.1 Research design

The research methodology adopted encompassed elements of both phenomenology and ethnography. Phenomenology focuses on the lived experiences of people and the way they construct their experiences and reality and is a useful way to understand social phenomena through interpretation (Kienzler, 1991; Smith, 2018). It facilitates deep and empathetic engagement with individuals, in order to understand how they give meaning to their lives and their “lifeworld” (Schutz et al., 1978). This was combined with ethnographic (observational) methods, which are well-suited to exploring people's actions in a given social context and their own interpretation of such behavior (Hammersly and Atkinson, 1983) and this included observations of activities and meetings and interviews with participants and community assets group leaders. This approach enabled researchers to develop a more contextual understanding of the relationship between wellbeing and community activities (Corrigan et al., 2020) as well as facilitating rapport and gaining the trust of community group leaders and members. Semi-structured interviews were conducted either face to face, or online when pandemic restrictions were in place. One hundred and thirty three interviews were conducted in total across the three waves of research: 42 for wave one, 36 for wave two and 55 for wave three (see Table 1). Where possible the same participants who took part in the initial wave also participated in the two subsequent waves. As is evident from Table 1, two new community assets (Essex Integration, a voluntary sector organization providing practical help and support refugees and Colchester Islamic Community Centre, a group aiming to meet the needs of local Muslims) were added to the first sample list, as we recognized that there was little representation of ethnically diverse participants in our original sample.

**Table 1:** Overview of the three waves of community assets research conducted between 2019 and 2022.

**Wave**	**Community assets**	**Total number of participants per wave**
Wave one: data collected 2019 from eight community assets (Corrigan et al., 2020)	• Dementia Café, Clacton • Teen Talk, Harwich • Clacton and District Indoor Bowls Club • Project NOVA (a regional charity supporting veterans) • DNA Networks Uniform Exchange, Colchester (providing the exchange of second-hand children's school uniforms for vulnerable families) • Friendship Group (self-funded group of members who meet weekly, share lunch and participate in various social activities) • Parent/toddler: Colchester. The club provides a weekly meeting space for toddlers (from birth to 3 years) led by a trained volunteer. • Multiple Sclerosis Support Group (peer support)	42 participants took part in qualitative interviews
Wave two: data collected 2020 from community ten assets during the pandemic (Corrigan et al., 2021). The wave of the study reveals how the COVID-19 pandemic affected members of the original Community Groups and the study participant cohort was also extended, to include two new Community Assets composed of minority ethnic populations.	• Community Assets as above, plus two additional groups • Fresh Beginnings (later known as Essex Integration) Colchester (A voluntary sector organization providing practical help and support refugees) • Colchester Islamic Community Centre. A group aiming to meet the needs of local Muslims.	36 participants took part in qualitative interviews this included 15 follow-up interviews with previous assets, 8 from Colchester Islamic Community Centre and Fresh Beginnings and 13 organizers or co-organizers of Asset Groups.
Wave three: data collected from nine community assets post-pandemic (Corrigan et al., 2023a,b) Building upon previous waves (one and two), this study provides longitudinal insight to explore effects on the health and wellbeing of members since the lifting of lockdown restrictions brought about by the pandemic and the cost-of-living crisis	• As above. • However, it was not possible to conduct interviews with the Multiple Sclerosis Support Group as the group had not reassembled during the data collection period	55 participants took part in qualitative interviews

### 2.2 Study setting, sampling, and data collection

A purposive sample of community assets in Tendring and Colchester were chosen in consultation with members of the project Steering Group, and information was also provided by the two local Community Volunteer Service organizations. The sample selection followed the brief to include participants who were availing themselves of a community asset, and to ensure those groups reflected the health and wellbeing vulnerabilities of communities within the geographical areas of Tendring and Colchester. Together with the Local Authority commissioners we developed a “vulnerability matrix” focusing on groups located in areas of social deprivation, and/or vulnerable in terms of health, disability, and age and/or intersectional characteristics that would be considered as potentially excluded or marginalized (see Table 1 for details of sample based on our vulnerability matrix). A charity supporting veterans was also included, as they make up 2.79% of the population of Colchester Borough Council (2020) and have poor access to mental health support. Given that the definition of a community asset can include a place, three of the groups (the MS Group, the Parent and Toddler Group and the Friendship Group) included were part of a “community halls partnership project” serving vulnerable parts of Colchester. This project enabled several community halls to develop new ways of working collaboratively by centralizing management and administration with the aim of forming a “network” of appropriate and sustainable community buildings.

To identify socially deprived areas we used official statistics (Ministry of Housing, Communities and Local Government, 2019), which measured relative deprivation at small-area levels in England. The organizational structure of the assets varied, and this was intentional in our sampling, as we wanted to find out the wellbeing benefits irrespective of group size, frequency of meetings, purpose, and funding sources.

To help identify assets we also used information previously gathered by the Community Volunteer Services in Colchester and Tendring who had mapped and identified community assets and we carried out online searches to find other community assets. We then approached the asset leaders about their co-operation in the study who then asked members if they would be willing to potentially participate in our research. The initial scope of the recruitment from wave one (Corrigan et al., 2020), was extended in waves two and three (Corrigan et al., 2021, 2023a,b) to increase the representation from minority ethnic participants (see Table 1).

### 2.3 Data analysis

All data was transcribed verbatim, except for the interviews with some members of Essex Integration, as the interviews were conducted in Farsi, and were translated by the interviewer using a “free translation” approach (Witcher, 2010). The transcriptions were analyzed using a thematic analysis approach, which involved identifying key topics and patterns, regularities, and contrasts in the data to create interpretive meaning (Braun and Clarke, 2006). The analysis was informed by issues and concepts that stemmed from the data, such as the identification of asset group members' resources (Antonovsky, 1987), as well as reading the data for “capabilities” that enabled some citizens to thrive (or not) in challenging situations (Nussbaum and Sen, 1993). To ensure internal validity of the data, the field researchers conducted initial coding and thematic analysis of the transcripts, and another member of the research team also read and analyzed a sample of transcripts. The findings were discussed further by the whole team.

### 2.4 Ethical considerations

Each wave of the study was granted ethical approved by Anglia Ruskin's University Research Ethics Panel and informed consent was recorded prior to interviewing and the ethnographic observations. Pseudonyms were used to protect the identity of participants.

## 3 Results

The collective findings from the research highlighted some common themes, and the research also informed a series of recommendations, and many of these were actioned within partnerships across the local ICS.

### 3.1 Sociality

One of the strongest themes to emerge from the research was the significance of sociality. Sociality is a variously defined term, but we use it here as a concept that captures the inherent nature of people to form social links, interact and engage in group activities while gaining pleasure in doing so. Across the three waves of the research, participants/asset members and leaders, highlighted the benefits of belonging to a community group and provided narrative accounts of the experiences of wellbeing that participants acquired through making social connections and bonds with others. By wellbeing we are referring to a broader concept than health that allude to a positive state shaped by subjective feelings and social experiences (Daykin et al., 2021). While there is no universally agreed measure of wellbeing, our analysis was based on narrative accounts of participants' lived experience and feelings such as feelings of happiness or anxiety, as well as on the perceptions of the extent of meaning and purpose associated with their experiences of the community asset (Daykin et al., 2021).

Participants revealed how social interactions, friendships, acts of kindness, the sharing of activities, food/refreshments and information improved people's feelings of wellbeing. A major benefit that members appreciated across all groups was how much community leaders, volunteers, and members genuinely cared for each other. The members of the different asset groups frequently reported that they felt “heard,” “understood,” “listened to.” and “valued.” Participants enjoyed being part of a group and expressed feelings of camaraderie with other members. Many participants regarded the community asset group they belonged to as a second family. During the first wave of research all participants were asked how important the asset was to them and their wellbeing. All described it as extremely important with many describing it as a “lifeline”.

### 3.2 Overcoming challenges

Another strong messages to emerge from the data was that the connections made within the community assets helped facilitate members' ability to cope with challenges they faced, with many members highlighting how their relationships with others helped them build resilience and maintain wellbeing. For some, the sense of belonging and connectedness found within the community assets helped them to recover from illness and manage their disability (Corrigan et al., 2023a,b).

Undoubtedly, pandemic lockdowns and the cost-of-living crisis were experienced as challenging for many participants. Community assets were valued more so during these periods of time. In many ways this finding is not unexpected, as social connectedness is now recognized as an important determinant of both physical and mental wellbeing (WHO, 2003; Cruwys et al., 2014). Furthermore, longitudinal research has found that membership of community groups and other organizations is strongly associated with positive mental health outcomes (Yu et al., 2015). However, what is distinctive about the research findings is the recognition of the role of community assets played in helping to maintain health and wellbeing through social connectedness irrespective of the function, size, funding, activity or frequency of meetings. Participants described how being part of a community asset and meeting up regularly, provided them with strength and resilience in the face of ongoing life challenges. Sharing with others who are facing similar situations and receiving support from group organizers also really helped most participants cope with adversity and build resilience.

### 3.3 Social isolation

Participants living alone and or experiencing challenges reported that meeting with others made them feel less isolated. Also, those living in places with poor transport infrastructure felt less isolated in communing together with others. Members of Teen Talk for example spoke of how coming together with like-minded peers helped overcome their negative feelings of living somewhere that was neglected and “left behind”, and the free transport provided to those attending the Dementia Café was highly valued by members who lived in remote areas and were no longer able to drive. Some members who traveled together in the minibus to the Dementia Café provided said how journey together lifted their moods.

Unsurprisingly, when the COVID-19 pandemic restrictions were implemented and group members were unable to meet, this had an extremely negative impact on participants' wellbeing. It was notable that those people who were isolated, living alone, and living some distance away from family, found the lockdowns extremely challenging (Corrigan et al., 2021). Many spoke of how they experienced feeling socially isolated. Social isolation is defined as a state in which “the individual lacks a sense of belonging socially, lacks engagement with others, has a minimal number of social contacts and is deficient in fulfilling and quality relationships” (Nicholson, 2009, p. 1346). Recent research has also identified elevated levels of anxiety and distress in communities during the pandemic, often related to anxiety of the unknown and the disruption of human connections (Xiong et al., 2020). Some participants experienced significant mental distress because of pandemic restrictions and not being able to meet with others. For example, one male member of the bowls club who lived on his own in a mobile home said that he experienced very bad depression and mental distress. Difficult emotions were more often described by those with a history of mental health problems and those who had experienced prior traumatic events (such as former soldiers, and refugees). Those living with difficult home situations, such as those living with a partner with dementia, also experienced very poor mental health while the pandemic restrictions were in place. Indeed, in some cases spouses of those with dementia experienced mental health breakdowns.

Most community assets were able to respond to the pandemic crisis by providing some form of remote support to members during the lockdowns, with some being quick to develop new forms of “remote” interaction when face to face contact was not permitted (e.g., meetings via zoom, telephone support and practical support such as dropping off food and medicine). Furthermore, most participants reported that they had some form of remote social contact from community asset leaders, other members, family, friends, and neighbors.

It was notable that the Colchester Islamic Community Centre excelled in its use of social media to communicate with members, although a few of the community assets were unable to offer a service or support members during lockdowns. Nonetheless, where community leaders reached out, this was greatly valued by participants, although smaller groups such as the friendship group, and the parent and toddler group, paused during lockdowns. However, most group leaders felt that the response that they were able to offer did not fully meet members' needs. This was the particularly the case with the Dementia café whose leaders reported that the welfare telephone calls they undertook were simply not sufficient to meet the distress of those living with dementia caused by lockdown restrictions. Prior to, and following the pandemic restrictions, participants living in less accessible parts of Tendring spoke of feeling isolated because of a lack of convenient and cheap transport. This was a particular problem for some older participants attending the Dementia Café who could no longer drive due to ill health and disability, as well the young adult members of Teen Talk who also spoke of Harwich as depressing and run down. This is not surprising, as Harwich is ranked in the top 10% of the most socially deprived areas of England (Ministry of Housing, Communities and Local Government, 2019).

### 3.4 Safe places

Listening to the members of the different groups, it was clear that the assets and activities took place in safe supportive environments, enabling participants to “be themselves” and many participants spoke of how “safe” and “welcome” they felt when attending the assets. These places were considered as inclusive, and many people spoke of how belonging to the asset boosted their self-confidence as they felt accepted and not judged. For example, one member of the parent and toddler group who self-identified as deaf, spoke of how she had, for the first time, experienced feeling so welcome and at home and not feeling like an outsider as she would usually experience in such social settings. Also, participants attending the free school uniform forums spoke of having to overcome initial feelings of shame and fear of stigma before first attending but how they soon felt at ease and welcome. Different participants offered numerous examples of ways in which friendships with members and asset leaders provided practical and emotional support and a sense of “belonging” (Corrigan et al., 2020, 2021, 2023a,b).

### 3.5 Information sharing

Most groups facilitated informal and formal modes of information sharing. People spoke and shared information with members about their experiences. Some groups had invited speakers on issues of common interest. It was interesting to note that some community assets also provided pathways of support enabling members to access public services. This “bridging role” was particularly apparent in community assets that were developed to meet the needs of minority ethnic communities (i.e., the refugee support group and the group for the Islamic community), who were sometimes less familiar with public sector services. It was also notable that during the pandemic lockdowns, many of the community assets were seen as a significant source of “trusted information”. For example, within the Colchester Islamic Community Centre many of the members and volunteers were medically trained, and they were able to offer culturally appropriate information and advice to the members during the pandemic (Corrigan et al., 2023a,b).

### 3.6 Activities

The research also identified a wide range of collective, physical and/or mental activities that groups engaged in Corrigan et al. (2020, 2021, 2023a,b). There is substantive evidence to show that physical activity can help to prevent some serious health conditions (Naci and Loannidis, 2013; Kyu et al., 2016; da Silveira et al., 2021). The indoor bowls club provided an opportunity for physical activity and many members spoke of the physical health benefits of playing bowls, as well as the positive effect that social interactions had on their mental health. Other groups also provided a wide range of opportunities for members, for example, Teen Talk linked members with other local charities and organizations to facilitate the learning of new skills, including gardening and sailing, as well as cookery and painting. The Bowls club and Teen Talk are in socially deprived towns and research indicates that many people from lower socio-economic groups are often unable to participate in physical activity (Sport England, 2018) and have limited access to sports facilities (Alliott et al., 2022) so this exemplifies the importance of such assets in supporting physical activity. Moreover, it has been suggested that when physical activity is located within local community groups, it often offers members an opportunity to collectively influence activities that meet their needs and priorities, rather than simply attending formal sports activities (Spaaij and Jeanes, 2013). Again, this was evident from the data as many participants spoke of how activities were provided at their request, and opportunities to try something new such as the sailing sessions with Teen Talk members or chair yoga for the MS group were greatly appreciated. In groups such as the friendship and MS groups where physical activities such as outings and exercise classes happened less frequently, participants said that just getting out of the house to attend meetings meant that they were more active. Members of the Friendship Group reported feeling physically and mentally better when they attended group meetings.

### 3.7 Volunteering

All the asset groups who took part in the study promoted volunteering and some assets such as the Bowls club were run entirely by volunteers, while others such as the refugee group, had a blended structure of paid staff and volunteers. It was notable that during the pandemic new volunteers often came forward in response to the changing needs of the asset group. For example, within the Colchester Islamic Community Centre, some people volunteered to help older members use the internet so that they could keep in touch and attend online activities, while other people volunteered to drop off food parcels and medicines to members, and masks for NHS workers and the wider community. Research shows that volunteering has a highly beneficial effect on the wellbeing of volunteers (Nichol et al., 2024). We found that some participants, particularly the volunteers who lead community assets, spoke of the sense of wellbeing they experienced in “giving back” to the community and helping others. However, some community assets experienced new challenges in recruiting volunteers in the post-pandemic setting. For example, a young person's community asset (Teen Talk) had relied heavily on volunteers prior to the pandemic. Many of their volunteers were older people offering to help young people with cookery and other skills but none of the 40 people who volunteered prior to the pandemic were willing or able to commit to volunteering in the aftermath. This experience is not unique to Essex, as other research has also highlighted that volunteering has fallen across the country (Dederichs, 2023). One of the recommendations we made was to provide support and training for volunteers.

### 3.8 Impact

The testimonies and voices of community assets members were presented to key stakeholders in a variety of forums including presentations given to working groups within the two Local Authority areas of concern. In addition to published written reports, Power Point presentations, other mediums of communication were deployed to promote study findings and recommendations. These included i-poems based on original transcripts that were produced as part of the initial study and a film of Bowls Club members that was produced to accompany the second report following their resumption of activities after lockdown (Corrigan et al., 2021). The dissemination was wide reaching, and coverage of the research was presented on BBC television news and local BBC radio (BBC News, 2021). The talk on BBC Essex radio program in October 2021 was part of a public phone in discussing the wellbeing benefits of belonging to a community group.

The three reports resulting from the different waves of research contained recommendations based on the findings and many of these were actioned within partnerships across the local ICS (Corrigan et al., 2020, 2021, 2023a,b). For example, following publication of wave one research findings, Colchester City Council (formerly Colchester Borough Council) began to develop a new strategy for communities in collaboration with voluntary and community groups, as well as statutory providers of services, under a new unified multi-agency umbrella group (ONE Colchester). They now work in partnership on significant issues affecting local communities, aiming to develop solutions that will lead to more resilient communities and secure lasting improvements to local life for future generations. It was reported at the time by the Chair of the Alliance that the findings would continue shape priorities in the future (Bryson, 2020). Moreover, the research findings from wave two and three (during and after the pandemic), contributed toward the growing understanding of the complexity of the health landscape and the multiplicity of actors who are able to promote health, beyond the traditional structures of public sector organizations in England. Presentations to different departments were given to both local government areas throughout the 3-year period of the research and the value of these assets as entities that support wellbeing was appreciated by those in attendance. Reporting back to the commissioners also led to additional funding allocation to support the on-going activities of some groups by the local government, as well as additional support and funding to Teen Talk by a local mental health charity. A workshop, jointly organized by the research team and members of North East Essex Health & Wellbeing Alliance, was held on completion of all three waves of research. More than 40 delegates from the local government offices, charities, community groups including some of the participating community assets attended to discuss the research findings and actions being taken to support communities. Workshop sessions included discussions on social prescribing activities within the region as well as funding and training support provision for community assets.

## 4 Discussion

The research undertaken undoubtedly illustrates the important positive role community assets play in supporting the wellbeing of members and those communities that assets support. Given that participants were vulnerable in terms of age, health, disability, ethnicity, or economic demographic circumstances, the support received by some individuals was life changing. Indeed, many described the community asset as a lifeline, and some went as far as to report that without it they would not be alive. Some participants reported that their experience of the support they received from the assets was in stark contrast to support (or lack of support) often experienced when they sought support from statutory services. Nevertheless, our final wave of research found that post-pandemic challenges and a cost-of-living crisis meant that many assets were under considerable strain. Ongoing UK Government spending cuts for public services following the pandemic and high rates of inflation and associated rises in the cost of living have left very many vulnerable populations more exposed. Some of the asset leaders we studied were experiencing a great increase in demand for their support. For example, the leader of the school uniform asset reported that the demand for free school uniforms had risen dramatically and that that there was a new demographic of working parents in non-deprived areas seeking support. Other assets previously mentioned such as the MS group and Teen Talk had been unable to resume full services since the pandemic. Also, the Dementia Café said that they were unable to support as many people as they had previously due to new policies on number of attendees since the pandemic. Some participants living in Tendring reported that public transport was reduced making them feel more isolated than before the pandemic.

### 4.1 Research and policy implications

Nevertheless, that this research was commissioned by the newly formed ICS organization, and the enthusiastic response by commissioners and local health and wellbeing staff who acted upon the studies' recommendations must be acknowledged. This research was part of these local government areas overall ABCD endeavors to support and listen to community voices and experiences. There is some indication therefore of a shift to a more complex, non-linear, collaborative approach to health and wellbeing indicative of complexity theory. The research reported here and elsewhere by Corrigan et al. (2020, 2021, 2023a,b) has highlighted the value of engaging community assets as partners in the co-production of local resources, and their value in contributing toward a therapeutic landscape that is part of a complex system that seeks to promote health and social inclusion.

This research represents a shift away from more positivistic approaches to population health that focus predominantly on epidemiological data and health interventions targeted at populations based on their behaviors, to one that recognizes social determinants of health and wellbeing and foregrounds narrative accounts based on people's lived experiences. We suggest that it can be considered an example of complexity theory insofar as it is a more complex approach that goes beyond the characteristics of the individual case and focuses on health as something which emerges from social environments (Byrne, 2002). We would recommend that further research be carried out to explore in more depth the relationship between community assets and complexity theory given the limited and tentative nature of this research.

While we welcome this more holistic approach, the current cuts in local government and healthcare systems that have followed a decade of austerity measures in the UK is undermining statutory services including funding to support ABCD. A recent report by the Institute for Government (2023) state cuts in public services are having a devastating impact with a record number of local government authorities going bankrupt and new NHS ICSs struggling to balance their budgets. Moreover, at a time when the public sector is subjected to deep fiscal cuts and reforms (Beatty and Fothergill, 2016; Carroll et al., 2021) community assets are at risk of being considered to be able to “fill the gap” in health and social care, despite often facing challenges in accessing funding and support themselves. Nonetheless, as Turnbull (2023) suggests, in this time of recession, community assets have the potential to be generative spaces that foreground “*everyday acts of care”*, where individuals and groups can “*share their experiences of navigating welfare bureaucracies”* and importantly, find friendship, companionship and a co-produced understanding of “living in place” in the wake of austerity.

## Data availability statement

The datasets presented in this article are not readily available because while the data is anonymous in its raw form it may be possible to identify individuals based on details revealed from the interview transcripts and we need to ensure anonymity to protect confidentiality as agreed with participants. Requests to access the datasets should be directed to oonagh.corrigan@aru.ac.uk.

## Ethics statement

The studies involving humans were approved by Faculty (of Health, Education, Medicine, & Social Care) Research Ethics Panel, Anglia Ruskin University. The studies were conducted in accordance with the local legislation and institutional requirements. The participants provided their written informed consent to participate in this study. Written informed consent was obtained from the individual(s) for the publication of any potentially identifiable images or data included in this article.

## Author contributions

OC: Conceptualization, Formal analysis, Funding acquisition, Investigation, Writing – original draft, Writing – review & editing. SDa: Writing – review & editing. SDo: Writing – review & editing. PL: Conceptualization, Writing – review & editing.

## References

[B1] Alliott O. Ryan M. Fairbrother H. van Sluijs E. (2022). Do adolescents' experiences of the barriers to and facilitators of physical activity differ by socioeconomic position? A systematic review of qualitative evidence. Obes. Rev. 23:13374. 10.1111/obr.13374 34713548 PMC7613938

[B2] Antonovsky A. (1987). The salutogenic perspective: toward a new view of health and illness. Advances 4, 47–55.

[B3] Apker J. (2012). Communication in Health Organizations. Malden: Polity Press.

[B4] BBC News (2021). Covid: Bowls Club Members in Essex Delighted to be Back With Friends. Available online at: https://www.bbc.co.uk/news/uk-england-essex-58875105#:~:text=Covid%3A%20Bowls%20club%20members%20in%20Essex%20delighted%20to%20be%20back%20with%20friends,-Published&text=Sports%20clubs%20and%20community%20groups,of%20their%20valued%20support%20networks (accessed April 29, 2024).

[B5] Beatty C. Fothergill S. (2016). The Uneven Impact of Welfare Reform: The Financial Losses to Places and People. Available online at: www.shu.ac.uk/centre-regional-economic-social-research/publications/the-uneven-impact-of-welfare-reform (accessed September 11, 2023).

[B6] Block P. (2018). Community: The Structure of Belonging. Oakland, CA: Berrett-Koehler Publishers.

[B7] Braun V. Clarke V. (2006). Using thematic analysis in psychology. Qual. Res. Psych. 3, 77–101. 10.1191/1478088706qp063oa32100154

[B8] Bryson R. (2020). Community Groups Set to Receive Further Support After Report. Colchester: Daily Gazette. Available online at: https://www.gazette-news.co.uk/news/18600018.community-groups-set-receive-support-report/ (accessed April 29, 2024).

[B9] Byrne D. (2002). Complexity Theory and the Social Sciences: An Introduction. London: Routledge.

[B10] Carroll A. Stokes D. Darley A. (2021). Use of complexity theory in health and social care: a scoping review protocol. BMJ Open 11:e047633. 10.1136/bmjopen-2020-047633 34321301 PMC8319978

[B11] Charles A. Ewbank L. Naylor C. Walsh N. Murray R. (2021). Developing Place-Based Partnerships. The Foundations of Effective Integrated Care Systems. The King's Fund. Available online at: https://www.kingsfund.org.uk/insight-and-analysis/reports/place-based-partnerships-integrated-care-systems (accessed April 20, 2024).

[B12] Colchester Borough Council (2020). Key Statistics for Colchester. Available online at: https://www.colchester.gov.uk/info/cbcarticle/?catid=colchester-statistics&id=KA-01631 (accessed September 11, 2023).

[B13] Corrigan O. Danielsen S. Hughes S. Lane P. Azizi F. (2023b). The Lived Experience of Members in a Post-Covid, Cost-of-Living Crisis Time: Community Assets in Colchester & Tendring. North East Essex Health & Wellbeing Alliance. Available online at: https://aru.figshare.com/articles/report/The_lived_experience_of_members_in_a_post-Covid_cost-of-living_crisis_time_Community_Assets_in_Colchester_Tendring/23696364/1 (accessed April 20, 2024).

[B14] Corrigan O. Danielsen S. Wali Haque H. Hughes S. Kabir R. Vinnakota D. (2021). Lessons From Lockdown: Community Asset Members' Experiences During the COVID-19 Pandemic. North East Essex Health & Wellbeing Alliance. Available online at: https://www.researchgate.net/publication/355411585_Research_Team_Lessons_from_Lockdown_Community_Asset_Members%27_experiences_during_the_COVID-19_Pandemic_2_Lessons_from_Lockdown_Community_Asset_Members%27_Experiences_during_the_COVID-19_Pandemic (accessed April 29, 2024).

[B15] Corrigan O. Hughes S. Danielsen S. Doherty S. Kabir R. (2023a). The impact of engaging with community groups: asset-based approaches and the lived experiences of socially vulnerable populations in the UK. Front. Public Health 11:1156422. 10.3389/fpubh.2023.1156422 37533527 PMC10393127

[B16] Corrigan O. Hughes S. Doherty S. Kabir R. (2020). Overcoming Barriers to Health and Wellbeing: Community Assets in North East Essex. North East Essex Health & Wellbeing Alliance. Available online at: https://www.researchgate.net/publication/344875356_Overcoming_Barriers_to_Health_and_Wellbeing_Community_Assets_in_North_East_Essex (accessed April 20, 2024).

[B17] Cruwys T. Haslam S. A. Dingle G. A. Haslam C. Jetten J. (2014). Depression and social identity: an integrative review. Pers. Soc. Psych. Rev. 18, 215–238. 10.1177/1088868314523839 24727974

[B18] Currie G. Dingwall R. Kitchener M. Waring J. (2012). Let's dance: organization studies, medical sociology and health policy. Soc. Sci. Med. 74, 273–280. 10.1016/j.socscimed.2011.11.002 22218227

[B19] Cyril S. Smith B. J. Possamai-Inesedy A. Renzaho A. M. (2015). Exploring the role of community engagement in improving the health of disadvantaged populations: a systematic review. Glob. Health Act. 8:29842. 10.3402/gha.v8.29842 26689460 PMC4685976

[B20] da Silveira M. P. da Silva Fagundes K. K. Bizuti R. B. (2021). Physical exercise as a tool to help the immune system against COVID-19: an integrative review of the current literature. Clin. Exp. Med. 21, 15–28. 10.1007/s10238-020-00650-3 32728975 PMC7387807

[B21] Daykin N. Mansfield L. Meads C. Gray K. Golding A. Tomlinson A.. (2021). The role of social capital in participatory arts for wellbeing: findings from a qualitative systematic review. Arts Health 13, 134–157. 10.1080/17533015.2020.1802605 32809907

[B22] Dederichs K. (2023). Volunteering in the United Kingdom during the COVID-19 pandemic: who started and who quit? Nonprofit Volunt. Sect. Q. 52, 1458–1474. 10.1177/08997640221122814

[B23] Essex County Council (2019). Joint Strategic Needs Assessment: Colchester Local Authority Profile. Available online at: https://data.essex.gov.uk/dataset/exwyd/essex-jsna-and-district-profile-reports-2019 (accessed April 29, 2024).

[B24] Goddard M. (2023). Integrated care systems and equity: prospects and plans. J. Integr. Care 31, 29–42. 10.1108/JICA-08-2022-0044

[B25] Greenhalgh T. Papoutsi C. (2018). Studying complexity in health services research: desperately seeking an overdue paradigm shift. BMC Med. 16:95. 10.1186/s12916-018-1089-4 29921272 PMC6009054

[B26] Hammersly M. Atkinson P. (1983). Ethnography: Principles in Practice. Tavistock: London.

[B27] Hill K. Webber R. (2022). From Pandemic to Cost of Living Crisis: Low-Income Households in Challenging Times. Jospeh Rowntree Foundation. Available online at: From-pandemic-to-cost-of-living-crisis-low-income-families-in-challenging-times-Full-report.pdf (michaelharrison.org.uk) (accessed March 23, 2024).

[B28] Holland J. (1995). Hidden Order: How Adaptation Builds Complexity. Reading: Addison-Wesley Publishing Company.

[B29] Howarth M. Griffiths A. Da Silva A. Green R. (2020). Social prescribing: a ‘natural' community-based solution. Br. J. Community Nurs. 25, 294–298. 10.12968/bjcn.2020.25.6.294 32496851

[B30] Institute for Government (2023). Comment: The Government has Abdicated Responsibility for Public Services. London.

[B31] Kauffman S. (1995). At Home in the Universe. New York, NY: Oxford University Press.

[B32] Kauffman S. A. (1993). The Origins of Order: Self-Organization and Selection in Evolution. Oxford: Oxford University Press.

[B33] Kienzler W. (1991). “What Is a Phenomenon? The Concept of Phenomenon in Husserl's Phenomenology,” in The Turning Points of the New Phenomenological Era. Analecta Husserliana (The Yearbook of Phenomenological Research), eds. A. T. Tymieniecka (Dordrecht: Springer), 34.

[B34] Kings Fund (2022). Integrated Care Systems Explained: Making Sense of Systems, Places and Neighbourhoods. Available from: https://www.kingsfund.org.uk/publications/integrated-care-systems-explained (accessed April 29, 2024).

[B35] Kyu H. H. Bachman V. F. Alexander L. T. Mumford J. E. Afshin A. Estep K.. (2016). Physical activity and risk of breast cancer, colon cancer, diabetes, ischemic heart disease, and ischemic stroke events: systematic review and dose-response meta-analysis for the Global Burden of Disease Study 2013. BMJ 354:i3857. 10.1136/bmj.i3857 27510511 PMC4979358

[B36] Long K. M. McDermott F. Meadows G. N. (2018). Being pragmatic about healthcare complexity: our experiences applying complexity theory and pragmatism to health services research. BMC Med. 16, 1–9. 10.1186/s12916-018-1087-6 29921277 PMC6008915

[B37] Marmot M. Bloomer E. Goldblatt P. (2013). The role of social determinants in tackling health objectives in a context of economic crisis. Public Health Rev. 35, 1–24. 10.1007/BF03391694

[B38] Michaelson J. Mahony S. Schifferes J. (2012). Measuring Wellbeing: A Guide for Practitioners. London: New Economics Foundation.

[B39] Ministry of Housing Communities and Local Government. (2019). English Indices of Deprivation 2019: Mapping Resources. Available online at: https://www.gov.uk/government/statistics/english-indices-of-deprivation-2019 (accessed April 29, 2024).

[B40] Mughal R. Thomson L. J. Daykin N. Chatterjee H. J. (2022). Rapid evidence review of community engagement and resources in the UK during the COVID-19 pandemic: how can community assets redress health inequities?. Int. J. Environ. Res. Public Health 19:4086. 10.3390/ijerph19074086 35409769 PMC8998387

[B41] Naci H. Loannidis J. (2013). Comparative effectiveness of exercise and drug interventions on mortality outcomes: meta epidemiological study. BMJ 347:f5577. 10.1136/bmj.f5577 24473061 PMC3788175

[B42] Nason R. (2023). Challenges of implementing complexity in Healthcare. Healthcare Mgmt Forum. 36, 368–372. 10.1177/08404704231191956 37544743 PMC10604384

[B43] NEE-Alliance (2020). Our Work. North East Essex Health and Wellbeing Alliance. Available online at: https://www.nee-alliance.org.uk/our-work/ (accessed April 29, 2024).

[B44] NHS (2019a). The NHS Long Term Plan–A Summary. National Health Service. Available online at: https://www.longtermplan.nhs.uk/wp-content/uploads/2019/01/the-nhs-long-term-plan-summary.pdf (accessed April 29, 2024).

[B45] NHS (2019b). The NHS Long Term Plan. Available online at: https://www.longtermplan.nhs.uk/wp-content/uploads/2019/08/nhs-long-term-plan-version-1.2.pdf (accessed April 29, 2024).

[B46] Nichol B. Wilson R. Rodrigues A. Haighton C. (2024). Exploring the effects of volunteering on the social, mental, and physical health and well-being of volunteers: an umbrella review. Voluntas 35, 97–128. 10.1007/s11266-023-00573-z 37360509 PMC10159229

[B47] Nicholson N. R. Jr. (2009). Social isolation in older adults: an evolutionary concept analysis. J. Adv. Nurs. 65, 1342–1352. 10.1111/j.1365-2648.2008.04959.x 19291185

[B48] Nurture Development (2024). ABCD Training and Resources. Manchester.

[B49] Nussbaum M. Sen A. (eds.). (1993). The Quality of Life. Oxford: Clarendon Press.

[B50] Russell C. (2020). Rekindling Democracy: A Professional's Guide to Working in Citizen Space. Eugen, OR: Wipf and Stock Publishers.

[B51] Schutz A. Grathoff R. Parsons T. (1978). The Theory of Social Action: The Correspondence of Alfred Schutz and Talcott Parsons. Bloomington, IN: Indiana University Press.

[B52] Smith D. W. (2018). “Phenomenology,” in The Stanford Encyclopedia of Philosophy, ed. E. N. Zalta Available online at: https://plato.stanford.edu/archives/sum2018/entries/phenomenology (accessed April 29, 2024).

[B53] Spaaij R. Jeanes R. (2013). Education for Social Change? A freirean critique of sport for development and peace. Phys. Educ. Sport Pedagog. 18, 442–457. 10.1080/17408989.2012.690378

[B54] Sport England (2018). Spotlight on Lower Socio-Economic Groups. Active Lives Adult Survey November 2016–17. Available online at: https://www.sportengland.org/research-and-data/research/lower-socio-economic-groups (accessed April 29, 2024).

[B55] Stutton J. (2018). Asset-Based Work With Communities. Research in Practice for Adults. Available online at: https://www.researchinpractice.org.uk/media/l2bl25h1/ripfa_leaders_briefing_asset-basedworkwithcommunities_web-1.pdf (accessed April 29, 2024).

[B56] Thirsk L. M. Clark A. M. (2017). Using qualitative research for complex interventions: the contributions of hermeneutics. Int. J. Qual. Meth. 16, 1–10. 10.1177/1609406917721068

[B57] Turnbull N. (2023). Austerity's afterlives? The case of community asset transfer in the UK. Geogr. J. 10.1111/geoj.12513

[B58] WHO (2003). “Unemployment,” in Social Determinants of Health: The Solid Facts, eds. R. Wilkinson, and M. Marmot (Geneva: World Health Organisation).

[B59] Witcher C. S. (2010). Negotiating transcription as a relative insider: implications for rigor. Int. J. Qual. Methods 9, 122–132. 10.1177/160940691000900201

[B60] Xiong J. Lipsitz O. Nasri F. Lui L. Gill H. Phan L.. (2020). Impact of COVID-19 pandemic on mental health in the general population: a systematic review. J. Aff. Dis. 277, 55–64. 10.1016/j.jad.2020.08.001 32799105 PMC7413844

[B61] Yu G. Sessions J.G. Fu Y. Wall M. (2015). A multilevel cross-lagged structural equation analysis for reciprocal relationship between social capital and health. Soc. Sci. Med. 142, 1–8. 10.1016/j.socscimed.2015.08.004 26277109

